# Valproic acid inhibits the invasion of PC3 prostate cancer cells by upregulating the metastasis suppressor protein NDRG1

**DOI:** 10.1590/S1415-475738420150028

**Published:** 2015

**Authors:** Jae Eun Lee, Jung Hwa Kim

**Affiliations:** Department of Biological Sciences, Inha University, Incheon, South Korea

**Keywords:** VPA, NDRG1, prostate cancer cells

## Abstract

Valproic acid (VPA) is a clinically available histone deacetylase inhibitor with promising anticancer attributes. Recent studies have demonstrated the anticancer effects of VPA on prostate cancer cells. However, little is known about the differential effects of VPA between metastatic and non-metastatic prostate cancer cells and the relationship between the expression of metastasis suppressor proteins and VPA. In the present study, we demonstrate that inhibition of cell viability and invasion by VPA was more effective in the metastatic prostate cancer cell line PC3 than in the tumorigenic but non-metastatic prostate cell line, RWPE2. Further, we identified that the metastasis suppressor NDRG1 is upregulated in PC3 by VPA treatment. In contrast, NDRG1 was not increased in RWPE2 cells. Also, the suppressed invasion of PC3 cells by VPA treatment was relieved by NDRG1 knockdown. Taken together, we suggest that the anticancer effect of VPA on prostate cancer cells is, in part, mediated through upregulation of NDRG1. We also conclude that VPA has differential effects on the metastasis suppressor gene and invasion ability between non-metastatic and metastatic prostate cancer cells.

## Introduction

Valproic acid (VPA, 2-propylvaleric acid) is a branched, short-chain fatty acid that has been widely used as an antiepileptic drug (Chateauvieux *et al.*, 2010). Recently, VPA has attracted a lot of attention due to its anticancer activity, which is thought to be mediated by histone deacetylase (HDACs) inhibition. VPA is a member of a promising new class of anticancer agents that affect proliferation, differentiation, and apoptosis in both solid and hematologic malignancies (Blaheta and Cinatl, 2002). Several studies have demonstrated the anti-tumoral characteristics of VPA in prostate cancer cells (Blaheta and Cinatl, 2002; Chateauvieux *et al.*, 2010). VPA diminishes cell proliferation and migration of prostate cancer cells by modulating multiple pathways, including cell cycle arrest, apoptosis, and angiogenesis (Gao *et al,.* 2007; Shabbeer *et al.*, 2007; Wedel *et al.*, 2011; Sidana *et al.*, 2012; Witt *et al.*, 2013). However, the association between the expression of metastasis suppressor genes and VPA has not been studied in detail.

Metastasis suppressor genes inhibit metastasis but do not affect the growth of primary tumors. The expression or function of metastasis suppressor genes is lost primarily in many metastatic cancers. Interestingly, restoration of metastasis suppressor gene expression could inhibit cancer metastasis (Smith and Theodorescu, 2009). Metastasis suppressor proteins participate in the regulation of multiple steps in the metastatic process, including cancer cell invasion, survival in the bloodstream, and survival at the secondary site (Smith and Theodorescu, 2009). The low expression of metastasis suppressor genes in highly metastatic cancers is dedicated to the epigenetic control and in some cases posttranslational regulation (Ballestar and Esteller, 2008; Ellis *et al.*, 2009). N-myc Downstream Regulated Gene-1 (NDRG1) is a metastasis suppressor gene, and its implication in cancer progression and metastasis has been extensively studied. The anti-metastatic function of NDRG1 as a metastasis suppressor protein has been identified in multiple cancers including breast, colon, prostate, and gastric cancers (Bandyopadhyay *et al.*, 2003, 2004; Maruyama *et al.*, 2006; Jiang *et al.*, 2010).

E-cadherin is a tumor suppressor which is highly expressed in epithelial cells and plays crucial roles in cell-cell adhesion. Downregulation of E-cadherin expression is the hallmark of the epithelial-to-mesenchymal transition (EMT) process, the mechanism by which immotile epithelial cells convert to the motile mesenchymal phenotype (Yang and Weinberg, 2008). Loss of E-cadherin expression or function is associated with cancer cell invasion and metastasis (Frixen *et al.*, 1991).

In this report, we aimed to investigate the differential effects of VPA on metastatic *vs.* non-metastatic prostate cancer cells and the relationship between the expression of metastasis suppressor proteins and VPA. We found that the metastatic prostate cancer cell PC3 was more sensitive to VPA treatment, regarding cell viability than the non-metastatic prostate cancer cell RWPE2. Furthermore, VPA induced the metastasis suppressor protein NDRG1 in PC3 cells but not in RWPE2. Finally, we found that induction of E-cadherin expression by VPA treatment was inhibited by NDRG1 knockdown. Moreover, when NDRG1 was knocked down the inhibition of PC3 invasion by VPA was relieved. We, hence, conclude that VPA might function more effectively on metastatic prostate cancer than on non-metastatic prostate cancer and that the anticancer effect of VPA on prostate cells is, in part, mediated by the induction of NDRG1.

## Methods

### Cell lines and Cell culture

PC3 cells were maintained in RPMI1640 medium (Welgene) supplemented with 10% FBS. RWPE2 cells were maintained in Keratinocyte-Serum Free Medium (K-SFM, Invitrogen) supplemented with 12.5 mg/L bovine pituitary extract (BPE) and 1.25 μg/L EGF. All cells were supplemented with an antibiotic-antimycotic solution (100 units/mL penicillin, 0.1 mg/mL streptomycin, and 0.25 mg/mL amphotericin B) and grown at 37 °C in standard cell culture conditions (5% CO_2_, 95% humidity).

### Antibodies

Antibodies were purchased from the manufacturers as follows: anti-NDRG1 (ab37897 and ab124689, Abcam), anti-BRMS1 (ab134968, Abcam), anti-NM23H1 (sc-56928, Santa Cruz) anti-E-cadherin (610181, BD Transduction Laboratories), anti-vimentin (sc-32322, Santa Cruz), anti-β-actin (A1978, Sigma-Aldrich).

### Real time RT-PCR and knockdown

Total RNA was extracted from PC3 treated with VPA (0, 0.75, 1, 3 mM) for 24 h using Trizol reagent (Invitrogen). Reverse transcription reactions were performed with 2 ug of total RNA using RevertAid M-MuLV reverse transcriptase (Thermo Scientific) and oligo (dT) primers (Fermentas) according to the manufacturer's protocol. The abundance of mRNA was detected by real-time quantitative RT-PCR using the ABI prism 7300 system (Applied Biosystems) and SYBR Green reagent (Molecular Probes). Transcript quantity of the NDRG1 gene was calculated using the ΔΔCt method by normalization to GAPDH. The measurement was performed in three independent biological experiments and each with three technical replicates. The sequences of the primer pairs were as follows: NDRG1 5′-CGCCAGCACATTGTGAATGAC-3′ and 5′-TTTG AGTTGCACTCCAC CACG-3′ (Chang *et al.*, 2013), and GAPDH 5′-CCACATCGCTCAGACACCAT-3′ and 5′-TGACAAGCTTCCCGTTCTCA-3′. The target sequence for small hairpin RNA for NDRG1 was 5′-AGACCACTCTCCTCAAGAT-3′. The primer containing the short hairpin RNA sequence targeting NDRG1 was annealed and cloned into a pMSCVpuro vector.

### Cell viability assay

PC3 and RWPE2 cells were plated at a density of 4 × 10^4^ cells per well in 6-well plates in duplicate, and after 24 h in clutlure they were treated with 0, 0.75, 1, 3 mM VPA. At the indicated time point after VPA treatment, viable cells were counted daily by a trypan blue-exclusion assay.

### *In vitro* invasion assay

A total of 2.5 × 10^4^ PC3 or RWPE2 stable cells were loaded onto the top of a 24-well Matrigel invasion chamber assay plate (BD Biocoat; BD Biosciences). RPMI1640 medium containing 15% FBS was added to the bottom chamber as a chemoattractant for PC3 cells, and K-SFM medium containing BPE and EGF and supplemented with 15% FBS was used for RWPE2 cells. After incubation for 22 h, the cells that had migrated to the lower surface of the filter were fixed with 100% methanol and stained with 0.5% Giemsa solution. Cells were counted in nine random fields per insert.

## Results

### Inhibition of prostate cancer cell cell viability by VPA

**Figure 1:**
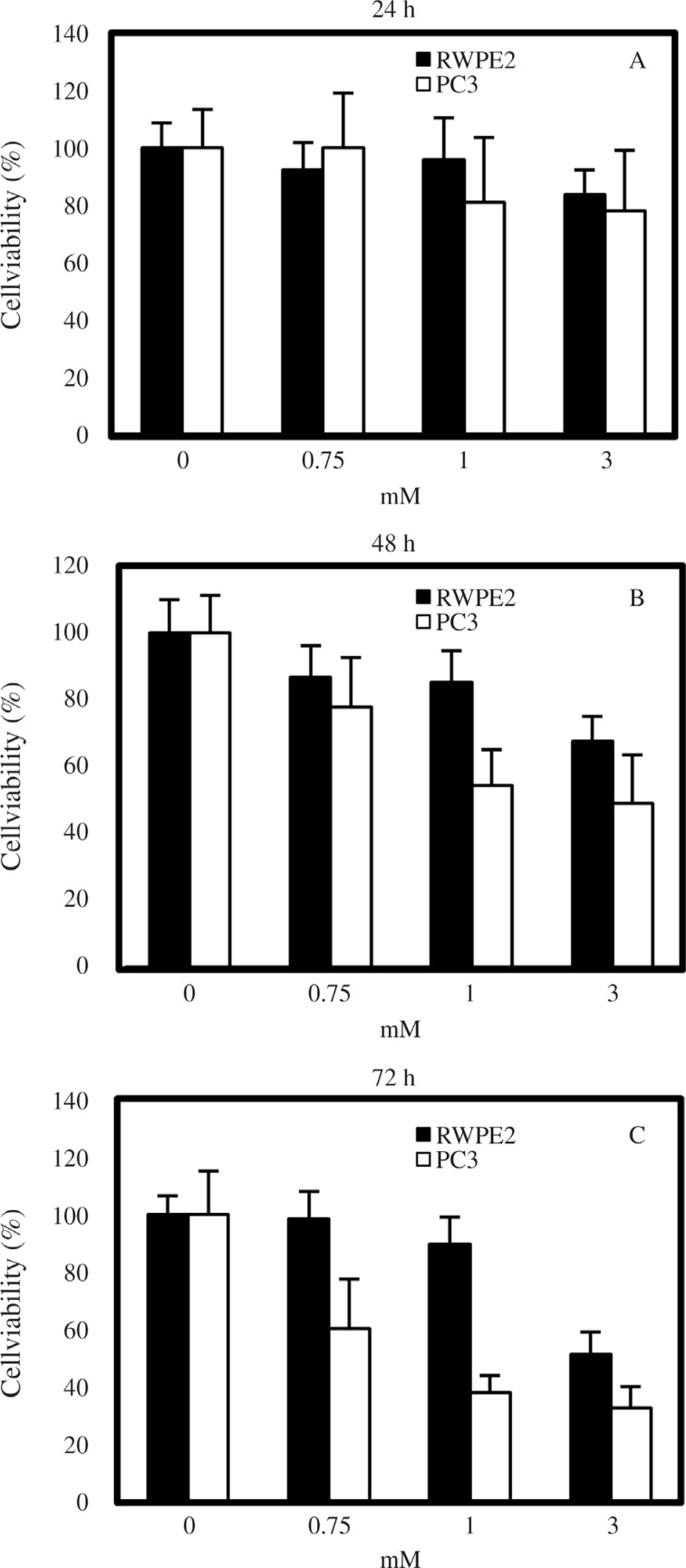
Effects of VPA on prostate cell viability. 24 h after cell seeding, PC3 and RWPE2 cells were treated with 0, 0.75, 1, 3 mM VPA. Viable cells were counted after 24 (A), 48 (B), and 72 (C) h of treatment. The results are expressed as % of viable cells compared to the control. Values are expressed as % cell viability ± SD of three independent experiments.

In the present study we aimed to determine the differential effects of VPA in relation to the metastatic potential of prostate tumor cells. To do so, the highly metastatic prostate cancer cell line PC3 and the tumorigenic but non-metastatic prostate cancer cell line RWPE2 were treated with VPA (0, 0.75, 1, and 3 mM) and the degree of cell viability was assessed by counting the viable cells. In both cell types, VPA inhibited cell viability in a dose-dependent fashion, and the duration of VPA treatment also had a positive relationship with cell viability inhibition (Figure 1). The effects of VPA on the viability of PC3 cells were in accordance with those of a previously published study (Xia *et al.*, 2006). Notably so, PC3 cells were more sensitive than RWPE2 cells to the viability inhibition activity of VPA. At the concentrations of 0.75 and 1 mM VPA, the viability of RWPE2 was not significantly decreased when compared to controls (0 mM VPA). However, after 72 h of treatment with 1 mM VPA, the viability of PC3 cells (38.2% ± 6.1%) was two-fold lower than that of RWPE2 cells (76.6% ± 8.0%). After 72 h treatment with 3 mM VPA, the viability of RWPE2 cells had also decreased by almost 50%, but was still higher than that of PC3 cells (32.9% ± 7.3%). This result indicates a considerable difference in the effects of VPA on cell viability between metastatic prostate cancer cells and non-metastatic cancerous prostate cells.

### Suppression of PC3 invasion by VPA treatment

**Figure 2:**
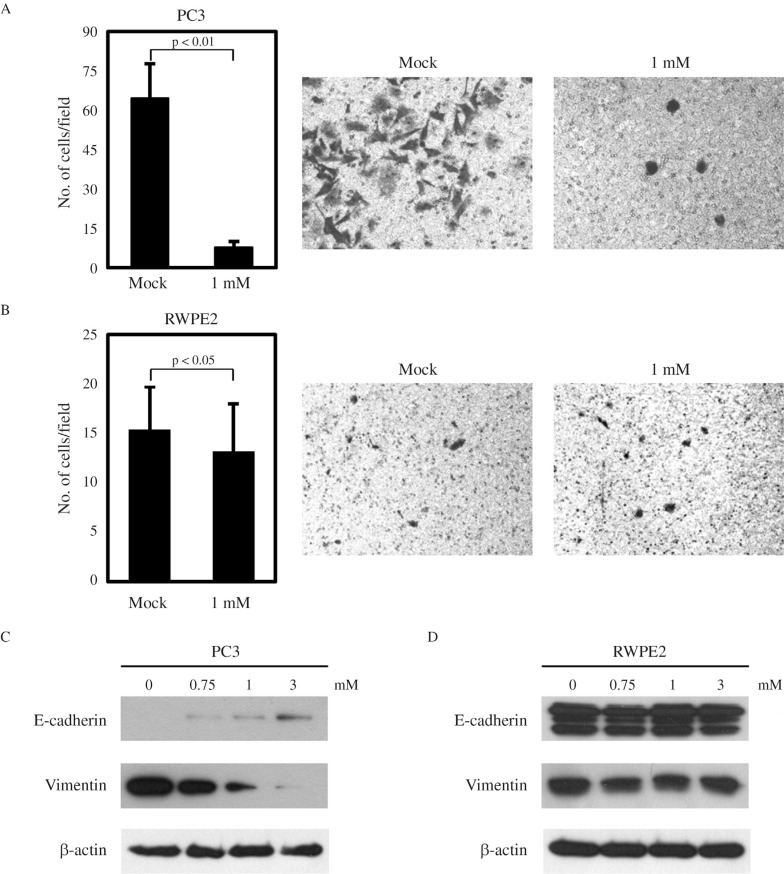
Effects of VPA on the invasiveness of prostate cells. Matrigel invasion assays for PC3 (A) and RWPE2 (B) cells. The same amount of each cell type was seeded onto the matrigel coated chamber and treated with 1 mM VPA. After 22 h, the invaded cells on the lower surface of the membrane were counted. Values are expressed as mean ± SD of three independent experiments, and the *p* value shown is from a Student's t-test analysis. The expression of E-cadherin and vimentin was examined by immunoblotting of PC3 (C) and RWPE2 (D) cells treated with VPA.

Next, we checked whether the invasive ability of prostate cancer cells is differentially regulated by VPA. PC3 cells have a high invasive potential, and VPA has been reported to significantly inhibit PC3 invasion (Wedel *et al.*, 2011; Jiang *et al.*, 2014) (Figure 2A). As expected, RWPE2 cells showed an extremely low invasive ability, and VPA treatment only slightly reduced their invasiveness. EMT is important for tumor cells to acquire migratory and invasive properties and allows tumor cells to infiltrate surrounding tissues and ultimately metastasize to distant sites (Yang and Weinberg, 2008). The hallmark of EMT is the change in expression of the epithelial marker E-cadherin, as well as of the mesenchymal marker vimentin (Lee *et al.*, 2006; Thiery and Sleeman, 2006). Thus, we examined the effects of VPA treatment on the expression of E-cadherin and vimentin. As shown in Figure 2C, VPA induced the molecular alterations characteristic of the reversion of EMT in PC3 cells, with E-cadherin being increased and vimentin decreased in a dose-dependent manner. In contrast, there was no change in the expression of E-cadherin and vimentin by VPA in RWPE2 cells (Figure 2D). Hence, we conclude that the inhibitory effect of VPA on cell viability and invasion is more effective in the metastatic prostate cancer cell PC3 than in the non-metastatic prostate cancer cell RWPE2.

### Upregulation of NDRG1 by VPA in PC3 cells

**Figure 3:**
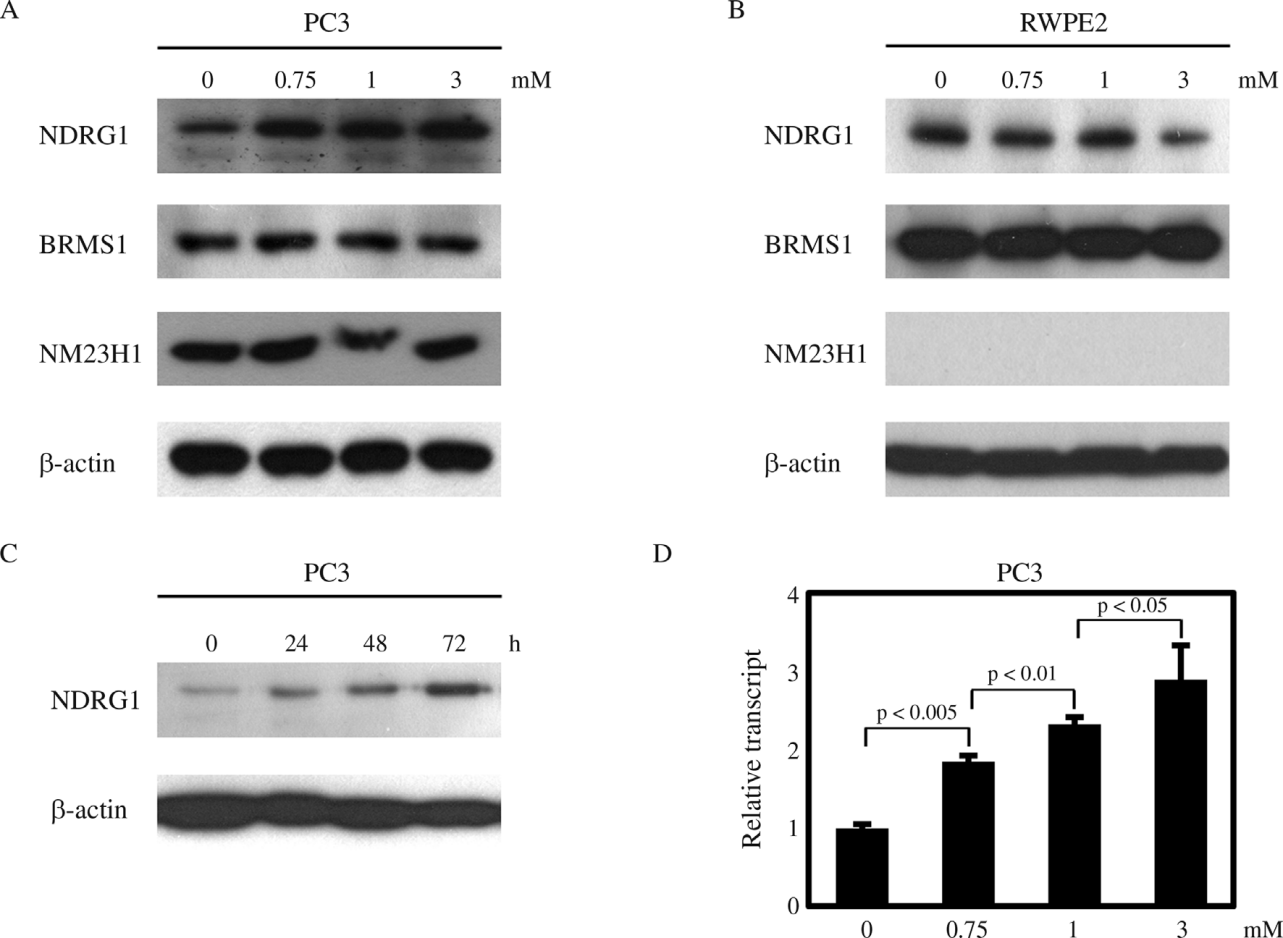
VPA increases NDRG1 expression in PC3 cells. 24 h after seeding, cells were treated with VPA. 24 h after VPA treatment, PC3 (A) and RWPE2 (B) cells were harvested for western blotting with NDRG1, BRMS1 and NME1 antibodies. (C) PC3 cells were treated with 1 mM VPA until the indicated time point. Protein levels of NDRG1 were monitored by western blotting. (D) Real-time quantitative RT-PCR analysis of NDRG1 after VPA treatment. Values are expressed as mean ± SD of three independent experiments, and the p value shown is from a Student's t-test analysis.

Next, we investigated the effects of VPA on the expression of several metastasis suppressors, including NDRG1, BRMS1, and NM23H1. Interestingly, NDRG1 was upregulated by VPA treatment in a dose-dependent manner in PC3 cells, while the expression of BRMS1 and NM23H1 was not affected (Figure 3A). In contrast, there was no increase in NDRG1 expression in RWPE2 cells treated with VPA (Figure 3B). Furthermore, in PC3 cells the up-regulation of NDRG1 was augmented when the duration of VPA treatment was prolonged (Figure 3C). Real-time PCR assays revealed that the NDRG1 transcript level was also increased by VPA in PC3 cells (Figure 3D). From these data, we hypothesize that VPA exerts its anti-tumor activity more specifically in metastatic prostate cells than in non-metastatic prostate cancer cells, in part, by upregulating NDRG1 expression in metastatic prostate cells.

### Implication of NDRG1 in VPA-mediated suppression of invasiveness

**Figure 4:**
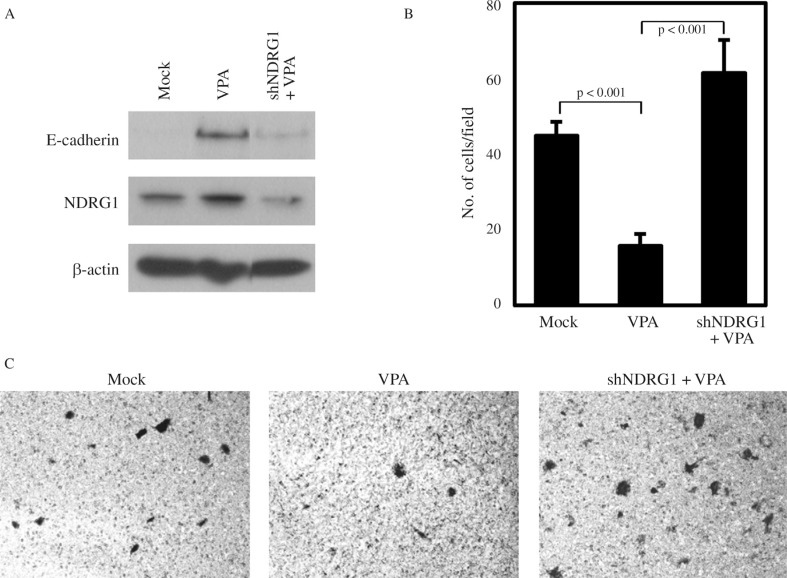
Knockdown of NDRG1 relieves the VPA-mediated suppression of PC3 invasiveness. (A) PC3 cells were transfected with NDRG1 shRNA or treated with 1 mM VPA for 24 h as indicated. Protein levels of E-cadherin and NDRG1 were monitored by western blotting. (B) The same amount of Mock vector control or NDRG1 shRNA transfected PC3 cells was seeded on a matrigel coated chamber and treated with 1 mM VPA. After 22 h, the cells that had invaded the lower surface of the membrane were counted. Values are expressed as means ± SD of three independent experiments, and the p value shown is from a Student's t-test analysis. (C) Microscopic image of matrigel invaded cells stained with Giemsa from the experiment shown in (B).

In prostate cancer cells, NDRG1 overexpression has been shown to maintain membrane E-cadherin and inhibit TGF-β-induced EMT (Chen *et al.*, 2012). Also, the involvement of NDRG1 in E-cadherin recycling and stabilization has been reported (Kachhap *et al.*, 2007). Thus, we investigated whether the increase of E-cadherin by VPA treatment in PC3 may be associated with VPA-mediated NDRG1 induction. We could show that the increase in E-cadherin caused by VPA treatment was inhibited by NDRG1 knockdown even in the presence of VPA (Figure 4A). Furthermore, the change in E-cadherin expression by NDRG1 knockdown was reflected in the invasiveness of PC3, as the suppressed PC3 invasion by VPA treatment was relieved by NDRG1 knockdown (Figure 4B,C). These results indicate that the anticancer activity of VPA in metastatic prostate cancer cells is in part mediated through the induction of the metastasis suppressor NDRG1.

## Discussion

In the present study we showed that VPA treatment has differential effects on metastatic *vs.* non-metastatic prostate cancer cells, with respect to cell viability and invasiveness, as well as expression of metastasis suppressor proteins. We found that highly metastatic PC3 cells were more sensitive to VPA treatment in the inhibition of cell viability and invasiveness than the non-metastatic RWPE2 cells. The inhibitory effects of VPA on cell viability and proliferation, as well as invasiveness of PC3 cells had already been investigated by other groups (Xia *et al.*, 2006; Iacopino *et al.*, 2008; Wedel *et al.*, 2011; Jiang *et al.*, 2014). Furthermore, previous reports had shown that the anti-invasive activity of VPA was valid only in PC3 but not in LNCaP prostate cancer cells with low metastatic potential (Annicotte *et al*,. 2006). This leads us to conclude that the anticancer activity of VPA is more effective on metastatic prostate cells than non-metastatic cells.

Epigenetic regulation of NDRG1 expression has been studied in some cancers. In the colon cell line SW620, several histone markers were related to the transcriptional repression of NDRG1, but this was not due to NDRG1 promoter methylation (Li and Chen, 2011). Treatment with 5-Aza-2′-deoxycytidine and Trichostatin A enhanced NDRG1 protein expression in pancreatic cancer cell lines, and again, there was no methylation of CpG island in the NDRG1 promoter (Angst *et al.*, 2010). Recently, it has been shown that promoter methylation of the NDRG1 gene was associated with reduced NDRG1 expression but not with histone modification in gastric cancer cells and tissue samples (Chang *et al.*, 2013). Thus, it seems that epigenetic modification of NDRG1 may vary among different cells and tissues. In this report we demonstrated that NDRG1 is upregulated by VPA in highly metastatic prostate cancer cells but not in non-metastatic prostate cancer cells. We also detected an increase in NDRG1 expression in another metastatic prostate cancer cell line DU145, but not in LNCaP (data not shown). This indicates that there are fundamental epigenetic differences between non-metastatic and metastatic prostate cancer cells that could alter the transcriptional response to VPA. Thus, we suggest that VPA can have a greater therapeutic benefit in treatments of metastatic prostate cells than non-metastatic cells, partly by up-regulating NDRG1. It has been reported that VPA inhibits MDA-MB-231 breast cancer cell migration by NM23H1 up-regulation (Li *et al.*, 2012). However, in our study we could not detect an increase in NM23H1 expression, neither in PC3, nor in RWPE2 cells. This means that the effects of VPA on gene expression may be tissue specific.

We found that VPA induces the molecular alterations characteristic of MET, which is the reverse process of EMT, in PC3 cells. E-cadherin was increased and vimentin decreased (Figure 2C). The up-regulation of E-cadherin expression by VPA seen in the invasive prostate cancer cells is consistent with previous reports (Annicotte *et al.*, 2006; Iacopino *et al.*, 2008; Zhang *et al.*, 2011). In contrast, VPA did not cause an E-cadherin up-regulation in non-metastatic prostate cells. EMT is important for tumor cells to acquire migratory and invasive properties and allows tumor cells to infiltrate surrounding tissue and ultimately metastasize to distant sites (Yang and Weinberg, 2008). It has been suggested that NDRG1 maintains the membraneous E-cadherin and regulates E-cadherin recycling (Kachhap *et al.*, 2007; Song *et al.*, 2010). Furthermore, upregulation of NDRG1 by iron chelators inhibits the TGF-β-induced EMT (Chen *et al.*, 2012). In the present study, we showed that the up-regulation of E-cadherin by VPA is partially mediated through the induction of NDRG1. The invasion inhibitory action of VPA in metastatic cells is, thus, apparently mediated through the metastasis suppressor NDRG1, and the restoration of metastasis suppressors by the action of the epigenetic modulator VPA could be a promising strategy for metastatic cancer treatment.

We consider that the differential anticancer action of VPA toward metastatic prostate cancer cells highlights its more beneficial therapeutic effects in the treatment of metastatic cancers. The involvement of the metastasis suppressor NDRG1 in the action of VPA gives certain clues on the mechanism of action of this versatile drug and should contribute to the discovery of other powerful epigenetic therapies based on the regulation of metastasis suppressors.
